# Improving Accurate Blood Pressure Cuff Allocation in Patients with Obesity: A Quality Improvement Initiative

**DOI:** 10.3390/healthcare9030323

**Published:** 2021-03-13

**Authors:** Victoria Eley, Aaron Khoo, Christine Woods, Andre van Zundert

**Affiliations:** 1 Department of Anaesthesia and Perioperative Medicine, The Royal Brisbane and Women’s Hospital, Herston 4006, Australia; christinea.woods@health.qld.gov.au (C.W.); vanzundertandre@gmail.com (A.v.Z.); 2 Faculty of Medicine, The University of Queensland, Herston 4006, Australia; aaron.khoo@uq.net.au

**Keywords:** blood pressure cuff sizes, mid-arm circumference, non-invasive blood pressure, obesity

## Abstract

Accurate noninvasive blood pressure (NIBP) measurement requires use of an appropriately sized cuff. We aimed to improve the perioperative allocation of NIBP cuffs in patients with Class II–III obesity. In the baseline evaluation, we measured the mid-arm circumference (MAC) of 40 patients with BMI > 35 kg/m^2^, documenting the corresponding cuff allocated by pre-operative nurses. The intervention consisted of the introduction of cuff allocation based on MAC measurement and augmented NIBP cuff supplies. We completed a re-evaluation and evaluation of the intervention by staff survey, using 5-point Likert scales and free text comments. At baseline, the correct cuff was allocated in 9 of 40 patients (22.5%). During the intervention, education occurred in 54 (69.2%) peri-operative nursing staff. Upon re-evaluation, the correct cuff was allocated in 30 of 40 patients (75.0%), a statistically significant improvement (χ^2^ = 22.1, *p* < 0.001). Ninety-three of 120 staff surveys were returned (78%). Eleven out of 18 preoperative staff surveyed (61.1%) felt confident measuring the arm and selecting the correct cuff. Six (33%) agreed that taking the arm measurement added a lot of extra work. Equipment shortages, accuracy concerns, and clinical workarounds were reported by staff. Our intervention increased the proportion of correct cuffs allocated, but equipment and practical issues persist with NIBP cuff selection in obese patients.

## 1. Introduction

**Figure 1:**
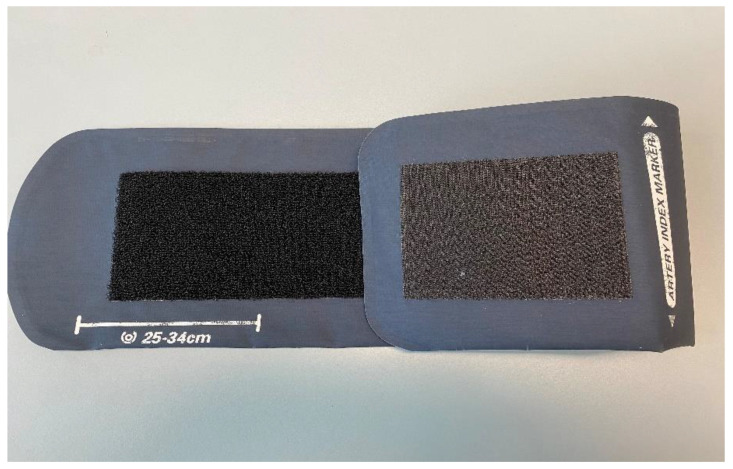
The Welch Allyn adult Size 11 cuff. The artery index marker should be situated between the white marker range (25–24 cm) once wrapped around the arm, to ensure correct sizing.

Accurate measurement of noninvasive blood pressure (NIBP) requires an appropriately sized arm cuff [1,2]. The size of the inflatable bladder within the cuff itself is required to have a length-to-width ratio of 2:1 [1], and the bladder length should encircle 80% of the arm circumference [2]. Manufacturers of blood pressure measurement devices provide a range of cuff sizes which have been validated in populations covering a specific range of arm circumferences [3]. The appropriate cuff may be selected based on a measurement of the mid-arm circumference (MAC; Table 1) or by using suggested markings provided on the blood pressure cuff by the manufacturer (Figure 1).

Patients with obesity may have arms of large circumference and are often of a conical shape, leading to challenges with blood pressure measurement [4,5]. The selection of correct cuff size is essential for accurate perioperative NIBP measurement in this high-risk population [6], who have a high burden of co-morbidities and tend to have poor perioperative outcomes [7]. Previously published audits have focused on the accuracy and maintenance of blood pressure measurement equipment [8,9]. These have focused on the accuracy of the NIBP measurement devices, rather than the correct allocation of NIBP cuff size. There is little published information on the allocation of NIBP cuffs to patients with obesity in clinical practice. In these patients, NIBP cuffs can be difficult to place on the upper arm. Adipose deposits result in a relatively short arm length, and the arm itself has been shown to be conical rather than cylindrical [1].

We initiated this quality improvement project to improve correct NIBP cuff selection in patients with a body mass index >35 kg/m^2^ presenting to our institution for elective surgery. The project was initiated after clinicians and researchers observed that incorrectly sized NIBP cuffs were being allocated to patients with obesity. Our aims are listed below.

1. Undertake a baseline evaluation.
2. Implement an intervention.
3. Re-evaluate practice.
4. Report the staff experience of the intervention.

## 2. Materials and Methods

This quality improvement initiative was undertaken at a single tertiary referral hospital in Brisbane, Australia. The human research ethics committee of the Royal Brisbane and Women’s Hospital (RBWH) approved an exemption from ethical review for the baseline evaluation (LNR/2020/QRBW/65216) and awarded ethical approval (with waiver of patient consent) for the intervention, re-evaluation of practice and staff surveys (LNR/2020/QRBW/66174). The 18 general operating theatres of the RBWH undertake all surgical sub-specialties other than cardiac surgery. Four operating theatres on the obstetric floor cater for obstetric, gynecological, and minor plastic surgery. Outpatients are prepared in the day-care unit at which time their reusable Welch Allyn (Welch Allyn Inc. New York, NY, USA) [10] NIBP cuff is allocated by pre-operative nursing staff. Prior to this project, NIBP cuff allocation was based on staff experience and observation of the arm size, together with the suggested markings on the Welch Allyn cuffs (Figure 1).

The allocated cuff is intended to accompany the patient intraoperatively (in the operating theatre) and post-operatively (in the post-anesthesia care unit (PACU)). At that point, it is removed from the patient, cleaned, and prepared for use again. The cuff may be changed at any time during the patient journey by the anesthesia medical staff.

### 2.1. Baseline Evaluation of Current Practice

At baseline we prospectively determined the proportion of patients with a BMI > 35 kg/m^2^ and presenting for surgery, who were allocated the correct NIBP cuff size, according to the manufacturer’s instructions (Table 1) [10]. For two weeks in July 2020, we documented the sex, BMI, and surgical specialty caring for the patients, and their right MAC was measured by trained personnel, according to approved anthropometric techniques [11]. We recorded data from 40 such patients, which, based on unpublished data from 2017, would comprise 67% of patients meeting those criteria. Author AK recorded the data and documented the size of the allocated cuff which was ascertained by direct observation. Based on the MAC measurement provided by AK, author VE determined the recommended cuff size according to the manufacturer [10]. There is overlap between the recommended cuff sizes, e.g., if the MAC was 33 cm, either an adult or large adult cuff could be allocated according to Table 1. When an “overlap” measurement occurred, VE determined that either size of cuff (the smaller or the larger option) was correctly allocated. Intraoperatively and post-operatively, we documented whether the NIBP cuff had been changed and if so, to what size. The proportion of patients allocated the correctly sized cuff in the intraoperative and post-operative period were considered secondary outcomes. We also documented if invasive arterial monitoring was utilized in either of these locations.

### 2.2. The Intervention

Having identified the extent of the problem, an interdisciplinary meeting was held, including pre-operative and PACU nursing leaders. This meeting aimed to discuss the current process for NIBP allocation. The discussion identified several potential areas for intervention: (1) Lack of awareness of the importance of cuff size. (2) Lack of awareness that the Size 13 (labeled as the “thigh cuff”) was the appropriate cuff for use on the arms of patients with a MAC of 40–55 cm (confirmed by advice from the company and consistent with American Heart Association recommendations [12]). (3) Allocation of cuffs based on methods other than measurement of MAC. (4) Lack of equipment for measuring the MAC. (5) Use of the visual guide on the NIBP cuff to select the appropriate cuff (Figure 1). (6) An assumption that the Size 11L cuff was suitable for patients with larger arms because it was labeled “long”. (7) A lack of the full range of NIBP cuff sizes in stock to allow for correct allocation at all times.

The intervention was designed to address these areas and consisted of three components:

1. Education of the pre-operative nurses allocating the NIBP cuffs and PACU nurses. This included face-to-face education via small-group sessions. This was undertaken by nursing educators, with supporting written education for circulation via email (Supplemental Figure S1). The group sessions and written education materials addressed:

- Ideal practice—how to measure the MAC and select the correct cuff?
- How to manage difficulties? For example, when the selected cuff does not provide a measurement.
- A recommendation to document the MAC measurement in the patient record, with the appropriate cuff size stated.

Laminated cards were distributed to the pre-operative, intraoperative, and postoperative care areas (Supplemental Figure S2). These cards showed the process for correct cuff allocation and listed the recommended NIBP cuff sizes with the related MAC ranges.

2. Provision of equipment: We ensured access to measuring tapes for measuring the MAC and augmented the current stock of NIBP cuffs, including more Size 12 and Size 13 cuffs. The number of pre-operative and PACU nurses who received the small group education was recorded and calculated as a proportion of the total nursing staff employed in these areas at the time.

### 2.3. Re-Evaluation of Practice

Three months following completion of the intervention, we repeated the baseline evaluation to determine the proportion of patients with a BMI > 35 kg/m^2^ and presenting for surgery, who were allocated the correct NIBP cuff size. The patient characteristics, MAC measurement, and allocated cuff size were determined in exactly the same way, by departmental research nurses and VE again determined the recommended cuff size. We also determined if the MAC measurement was documented in the patient record, if the allocated cuff had been exchanged for a different cuff (of inappropriate or appropriate size) in the intraoperative or post-operative stages of care, and whether an invasive arterial catheter had been utilized.

### 2.4. Evaluation of the Intervention—Staff Surveys

Over a period of one week (during the same period of re-evaluation), we sought feedback on the intervention from staff working three stages of care—pre-operative nursing staff, specialist anesthetists, and PACU nursing staff. The survey of the pre-operative staff evaluated staff experiences and sought to identify barriers to implementation of this process, using 5-point Likert scales (Supplemental Figure S3) and free text comments. Surveys of the specialist anesthetists and PACU nursing staff were identical. We evaluated whether the allocated cuff was usually acceptable or if it often needed to be changed, using 5-point Likert scales (Supplemental Figure S3) and free text comments. Completion of the questionnaires was voluntary with responses de-identified. Paper surveys were distributed and returned via an anonymous drop-box, or directly to the audit team. The proportion of staff responding was calculated from the known numbers of staff working in each area at that time.

### 2.5. Data Analysis and Outcomes

The primary outcome was the difference between the proportion of patients who were allocated the correct NIBP cuff size, assessed before and after the intervention. Secondary outcomes were staff survey responses and free text comments. Patient characteristics were described using mean (SD), median (IQR), and number (%). Responses to the survey Likert scales were presented as number (%) and as frequency histograms. The primary outcome was assessed using Chi-Square analysis with significance set at *p* < 0.05. Study data were collected and managed using REDCap electronic data capture tools hosted at Metro North Hospital and Health Service [13,14]. Analysis was undertaken using Microsoft Excel 365.

Free text comments were organized into concepts and themes by two of the investigators (VE and CW) using conventional content analysis [15]. Each investigator individually read and re-read the comments. After initial coding, the themes and concepts were identified by consensus and direct quotes used to illustrate the themes.

## 3. Results

### 3.1. Baseline Evaluation of Current Practice

The 40 patients had a median (interquartile range (IQR)) BMI of 41.6 (37.1–46.2) kg/m^2^. The minimum BMI was 35.1 kg/m^2^ and the maximum was 68.5 kg/m^2^. The mean (SD) right MAC was 40.0 cm (4.1) and the range was 35–52 cm. The surgical specialties caring for the patients are summarized in Table 2. Nine patients had the correctly sized NIBP cuff allocated (23%) (Table 3). Of the remaining 31 patients, all were allocated a cuff that was too small. No Size 13 cuffs were allocated, despite eight patients (20%) meeting the MAC for that size.

During the intraoperative period, nine of the 31 incorrectly allocated cuffs (29%) were changed by anesthesia staff from Size 11 to the appropriate Size 12. Therefore, intraoperatively the correctly sized cuff was used in 18 patients (45%). There were no cuff size changes between the intraoperative and PACU stages of care. In six patients (15%), only invasive arterial monitoring (not NIBP) was used in the intraoperative period and in five of those patients only invasive monitoring was also used in the PACU stage.

### 3.2. The Intervention

Small group education sessions were attended by 54 (69%) pre-operative and PACU nursing staff. Written education materials were distributed to all pre-operative and PACU nursing staff via staff email groups.

### 3.3. Re-Evaluation of Practice

The 40 patients had a median (IQR) BMI of 43.9 kg/m^2^ (39.7–47.5) and the range was 35.0–67.0 kg/m^2^. The mean (SD) right MAC was 39.8 cm (6.0) and the range was 29.5–57.0 cm. Thirty patients had the correctly sized cuff size allocated (75%), and this was a statistically significant change compared to the baseline evaluation (χ^2^ = 22.1, *p* < 0.001, Table 3). Of the 10 (25.0%) incorrectly allocated cuffs, six were too large and four were too small. Size 13 cuffs were allocated appropriately to all 11 patients requiring that size. In 21 (53%), the arm measurement and appropriate cuff size were documented in the medical record.

During the intraoperative period, three patients (7.5%) did not use a NIBP cuff as invasive arterial monitoring was used instead. Four NIBP cuffs were changed for intraoperative care; three to the correct, larger Size 13; and one to the incorrect smaller Size, 12. The latter case was a caesarean section and the Size 13 cuff was changed to facilitate the mother holding their neonate. Therefore, intraoperatively the correctly sized cuff was used in 33 of 36 patients (91.7%). This was a statistically significant increase compared to the baseline evaluation (χ^2^ = 9.3, *p* = 0.002).

### 3.4. Evaluation of the Intervention—Staff Surveys

Of the 120 surveys distributed, 93 were returned with an overall response rate of 78%. Of the 93 surveys, 18 (19%) were received from pre-operative nursing staff, 39 (42%) from specialist anesthetists, and 36 (39%) from PACU staff. Survey responses of the pre-operative nursing staff are shown in Table 4. Twelve (67%) of the pre-operative nurses agreed or strongly agreed that there was always a tape measure available to use. Five (28%) indicated that the correctly sized cuff was always available. Nine (50%) agreed that it was easy to measure the arm length and MAC, and 11 (61%) agreed or strongly agreed that they felt confident taking the measurement and choosing the correct cuff. Six (33%) agreed that taking the measurement added a lot of extra work.

Of the 39 specialist anesthetists surveyed, 28 (72%) agreed or strongly agreed that the allocated cuff was usually acceptable and only 10 (26%) agreed or strongly agreed that they often needed to change the cuff (Table 5). Of the 36 PACU nursing staff surveyed, 20 (56%) agreed or strongly agreed that the allocated cuff was usually acceptable and 18 (50%) agreed or strongly agreed that they often needed to change the cuff (Table 5).

Free text comments were provided by 20 staff; 2 from pre-operative nurses, 10 from specialist anesthetists and 8 from PACU nurses. Content analysis revealed 3 major themes which are presented in Table 6, with related concepts and deidentified quotes.

## 4. Discussion

Our intervention, consisting of nursing staff education and provision of equipment, increased the proportion of patients with obesity being allocated the correct NIBP cuff for use in the perioperative period. Following our intervention, the allocation of the Size 13 cuff improved from zero to 100% of patients in whom it was the appropriate cuff, based on their MAC measurement. Prior to our intervention, many patients with obesity were allocated an inappropriately small cuff whereas after our intervention this occurred less often. As part of our intervention, pre-operative nurses were required to allocate a NIBP cuff based on arm measurement and they perceived that this increased their workload. However, the majority of nurses felt confident obtaining the MAC measurements and a measuring tape was available to most staff. The free text comments provided by surveyed staff identified ongoing problems with the availability and suitability of equipment. Staff expressed concerns about the accuracy of measurements and identified clinical workarounds undertaken, when NIBP measurement in patients with obesity is challenging.

The importance of cuff size in the measurement of blood pressure has been known since Riva Rocci developed the method of auscultatory sphygmomanometry [16]. An inappropriately small cuff relative to the arm will tend to overestimate the true blood pressure, whereas an inappropriately large cuff will tend to underestimate the true blood pressure [17,18]. The American Heart Association provides recommendations for the dimensions of the inflatable bladder (inside the cuff itself) which are required for accurate measurements [12]; however, these may differ from those provided by manufacturers [10]. As the prevalence of obesity has increased worldwide [19], there is increasing acknowledgement of the practical difficulties presented by NIBP measurement in patients with obesity, due to the size and shape of their arms [20]. We have previously demonstrated that NIBP measurement may also be a negative experience for patients with obesity [4].

Prior to our intervention, pre-operative nursing staff allocated NIBP cuffs based on their experience and observations, and by making use of the markings on the cuff [10]. Our project has demonstrated that allocation based on MAC measurement resulted in more accurate allocation of NIBP cuffs. There was previously uncertainty as to the use of the Size 13 cuff, which is labelled as a “Thigh” cuff. However, this cuff is recommended for patients who are not suitable for the large adult size. Use of the Size 13 cuff increased following our intervention. Free text comments from our respondents show that even when the correct cuff is chosen, the cuff may not fit the arm shape. In these situations, the authors suggest that the allocation of the Size 13 cuff may act as a signal, that this individual has arms at the very high end of the MAC range and invasive monitoring, or other clinical workaround may be required. It is likely that clinician decision-making would be assisted, by MAC measurement documentation in the medical record. This aspect of care could be improved, based on our results. National data from the United States suggest than 9.5% of adults would require the Welch Allyn Size 13 cuff [21], whereas in 100 Australian patients presenting for bariatric surgery, 40% required the Size 13 cuff [4]. Thus, clinical decision-making by nurses and anesthetists concerning NIBP measurement in these patients commonly required.

In this project staff report concerns about the accuracy of NIBP readings in patients with obesity, as well as uncertainty about the course of action when the NIBP cuff does not fit or does not provide a reading. One anesthetist suggested they preferred the use of invasive arterial monitoring in patients with obesity, and this was reflected in our re-evaluation results, where three patients did not have any NIBP measured intraoperatively, only invasive monitoring. The intraoperative period presents many challenges, including drug-induced changes in blood pressure and heart rate [22], positioning requirements such as steep Trendelenburg or reverse Trendelenburg, as well as surgical interventions such as pneumoperitoneum [23]. All these factors result in rapid and somewhat unpredictable changes in blood pressure that must be detected accurately, in order to be managed appropriately. While invasive monitoring is feasible intraoperatively in patients with obesity, this is not an option in the pre-operative stage, or after discharge from the post-anesthesia care unit.

NIBP measurement in patients with obesity is an increasingly recognized problem and alternatives have been sought for accurate noninvasive measurements in this population [24,25,26]. Respondents in our study identified cuff placement on the forearm or leg as a clinical workaround, when placement on the arm was problematic or impossible. Studies evaluating the accuracy of forearm placement report conflicting results [27,28,29,30]. Recently a study evaluating intraoperative blood pressure measurements in patients with obesity suggested that NIBP measurements taken on the forearm were more accurate than those taken from the arm [31]. A major limitation of the latter study was the standard use of a large adult cuff in all patients [31], rather than use of a correctly sized cuff, based on MAC measurement and this compromises the results. Intraoperative hypotension, and to a lesser extent hypertension, are associated with increased perioperative morbidity and mortality [24]. Therefore, correct cuff selection, ensuring accurate NIBP measurement, is essential in patients with and without obesity. STRIDE BP is a group of international experts targeting both equipment and measurement technique in an effort to improve the accuracy of NIBP measurement in the home and in clinical contexts [32]. A proposed universal validation protocol for NIBP measurement devices [33] will ensure that devices are validated for specific MAC measurements, a feature not present in current validation protocols [34]. In the meantime, accurate NIBP devices that are not compromised by the size and shape of the arm are needed [31,35].

Our project has limitations. As this study was undertaken at a single center and tailored to the processes of our institution, our results may not be generalizable to other institutions. We have only assessed the application of one brand of NIBP cuffs. While our overall staff survey response rate was acceptable, pre-operative nursing staff were underrepresented in the group of respondents and we did not identify if they were the same staff who had completed training. The surveys were only administered following the intervention, rather than before and after the intervention and we have not identified which component of the intervention was effective. The completion of the staff survey was voluntary and therefore open to responder bias. The presence of our team undertaking the re-evaluation may have influenced staff behavior and affected our results. While we have demonstrated an improvement in the allocation and use of the correctly sized NIBP cuff in patients with obesity, we have not demonstrated more accurate NIBP measurements.

## 5. Conclusions

NIBP measurement is considered a core skill of nursing and medical staff. Our initial results demonstrated that in patients with obesity, the application of this core skill was compromised. By introducing the practice of cuff selection based on MAC measurement and augmenting the existing stock of all cuff sizes, we demonstrated an increase in correct NIBP allocation. Our project has highlighted the practical difficulties of applying recommended NIBP cuff sizes in patients with obesity. There remains room for improvement and it is important to acknowledge that technical difficulties persist, even when the correct NIBP cuff is allocated. Invasive arterial monitoring and clinical workarounds such as forearm cuff placement are sometimes required to achieve perioperative blood pressure measurement in patients with obesity. The evidence to support the accuracy of such workarounds is inconclusive. Future research must focus on the provision of alternative NIBP measurement devices for patients with obesity, which are accurate and not dependent on the size and shape of the arm.

## Figures and Tables

**Table 1:** Recommended noninvasive blood pressure cuff size as determined by mid-arm circumference and recommended by Welch Allyn [7].

Mid Arm Circumference Range (cm)	Recommended Cuff Size
25–34	Adult Size 11
32–43	Large Size 12
40–55	Thigh Size 13 (used on arm)

**Table 2:** Surgical specialties involved in the care of patients at baseline (*n* = 40) and re-evaluation (*n* = 40).

Baseline Evaluation *n* = 40		Re-Evaluation *n* = 40	
Surgical Specialty	*n* (%)	Surgical Specialty	*n* (%)
Bariatric surgery	7 (17.5)	Gynecology	18 (45)
Plastic surgery	6 (15)	Bariatric surgery	6 (15)
Gynecology	5 (12.5)	Plastic surgery	3 (7.5)
Ophthalmology	5 (12.5)	Obstetrics	3 (7.5)
Urology	4 (10)	Orthopedics	2 (5)
Ear, nose, and throat surgery	3 (7.5)	Ophthalmology	2 (5)
Neurosurgery	3 (7.5)	Other ^b^	6 (15)
Other ^a^	7 17.5)		

^a^. General surgery 1, obstetrics 2, burns 1, maxillofacial 1, orthopedic 1. ^b^. Vascular 1, ear nose and throat 1, urology 1, neurosurgery 1, ophthalmology 1, gastroenterology 1.

**Table 3:** The primary outcome: number and proportion of correct NIBP cuff size allocated before and after the intervention.

	Baseline Evaluation *n* = 40		Re-Evaluation *n* = 40		
	Number (%)		Number (%)		*p*-Value
Correct cuff size allocated	9	23	30	75	<0.001

**Table 4:** Statements and Likert scale responses from 18 pre-operative nurses following implementation of the intervention. Expressed as number (percent).

Statement	Strongly Disagree	Disagree	Neither Agree Nor Disagree	Agree	Strongly Agree
There was always the correct cuff size available when I needed it (12 responses).	0	4 (42)	2 (17)	4 (33)	1 (8)
It was easy to measure the arm length and mid arm circumference (12 responses).	0	1 (8)	2 (17)	5 (42)	4 (33)
I feel confident taking the measurement and choosing the correct cuff (12 responses).	0	0	1 (8)	8 (67)	3 (25)
Taking the measurements added a lot of extra work for me (11 responses).	0	3 (27)	2 (18)	6 (55)	0

**Table 5:** Statements and Likert scale responses from specialist anesthetists and PACU staff following implementation of the intervention. Expressed as number (percent).

	Responses from 39 Specialist Anesthetists				
Statement	Strongly Disagree	Disagree	Neither Agree Nor Disagree	Agree	Strongly Agree
The allocated blood pressure cuff is usually acceptable to me.	1 (3)	6 (15)	4 (10)	25 (64)	3 (8)
I often need to change the cuff to a different size.	4 (10)	17 (44)	8 (21)	8 (21)	2 (5)
	**Responses from 36 Post-Anesthetic Care Unit Nurses**				
The allocated blood pressure cuff is usually acceptable to me.	0	10 (28)	6 (17)	19 (53)	1 (3)
I often need to change the cuff to a different size.	0	14 (39)	4 (11)	16 (44)	2 (6)

**Table 6:** Results of conventional content analysis, responses from 20 staff concerning the implementation of NIBP cuff selection based on arm measurement. Pre-operative nurses: 2; intraoperative specialist anesthetists: 10; post-anesthetic care unit nurses: 8.

Theme	Concepts	Example
Problems with equipment	Availability: A range of NIBP cuff sizes are required and not always available	“it depends on if I can locate the right cuff” (pre-operative nurse)
	Suitability: The appropriate cuff suggested by arm measurement, does not fit the arm.	“the maroon cuffs (are) too wide for most upper arms” (post -anesthetic care unit nurse)
Accuracy of measurements in patients with obesity	Concern that non-invasive measurements are inadequate in patients with obesity.	“I am not happy with NIBP in place of Art-line” (specialist anesthetist)
		“I notice if there are problems in BP cuff or (the) reading is unexpected” (post-anesthetic care unit nurse)
Clinical workarounds when measurements are difficult		“sometime forearms and calves are used when they can’t get a BP on the patient’s arm” (post-anesthetic care unit nurse)
		“for patients with large upper arms and normal forearms I prefer to use a long standard cuff for a forearm BP” (specialist anesthetist)

## Data Availability

The data presented in this study are available on request from the corresponding author.

## Supplementary Materials

The following are available online at https://www.mdpi.com/2227-9032/9/3/323/s1. Figure S1: Teaching Materials, Figure S2: Laminated cards, Figure S3: Staff Questionnaires
