# Supplementary material for: Improving Accurate Blood Pressure Cuff Allocation in Patients with Obesity: A Quality Improvement Initiative
Source: Healthcare (Basel). 2021 Mar 13;9(3):323. doi: 10.3390/healthcare9030323 (PMC8000816; doi:10.3390/healthcare9030323)
Supplement: Supplementary file 1 [file healthcare-09-00323-s001.zip › S1 Teaching Material.pdf]

Teaching points – Correct Cuff Selection in Obese Patients.

What do we know?

Accurate blood pressure measurement relies on use of the appropriately sized cuff and this is based on the mid arm circumference.

Information from RBWH 2020

In obese patients (BMI > 35) only 9 out of 40 were allocated the correct cuff size.

None of these patients should have been allocated a blue cuff (20 did)

8 of these patients should have been allocated a black cuff (none did)

New initiative for obese patients, BMI >35 – measure the arm to select the cuff

1. Measure the arm according to the diagram provided.
2. Select the correct cuff size based on the arm measurement
3. Document on the check-in sheet (free text area).

Frequently asked questions

1. The black/size 13 cuff says it is a "thigh cuff" – should it be placed on the arm?

Yes. It is intended for use in people with very large arms.

2. Sometimes I can't get the black cuff to fit on the arm – what do I do then?

We understand that this is difficult and there is currently no good solution. Please document the arm measurement and the correct cuff. Sometimes the next best option is the maroon cuff on the arm or forearm, but the anaesthetist would like to know the correct size.

3. Sometimes I can't get a reading with the maroon or large cuffs.

Please document the arm measurement and the correct cuff and handover to the anaesthetic team/holding bay by writing in the free text section of the check-in sheet.

4. Sometimes I can't access the correct cuff size because there are none (I can't find them anywhere!!!). What do I do then?

Please document the arm measurement and the correct cuff size and provide what you can, and handover to the anaesthetic team/holding bay by writing in the free text section of the check-in sheet.

Please be assured that we will be asking for your feedback on the implementation of this new process.
