# Supplementary material for: Improving Accurate Blood Pressure Cuff Allocation in Patients with Obesity: A Quality Improvement Initiative
Source: Healthcare (Basel). 2021 Mar 13;9(3):323. doi: 10.3390/healthcare9030323 (PMC8000816; doi:10.3390/healthcare9030323)

S2. These were shown both sides of a laminated card for distribution in clinical areas.

Range (cm)	Size
25 – 34	ADULT LONG 11L
32 – 43	LARGE ADULT LONG 12L
40 – 55	THIGH 13 (USED ON ARM)

1. Measure arm length from the top of the shoulder to the tip of the elbow

2. At the halfway point of the arm length, measure the mid-arm circumference

3. Select the cuff using your measurement

4. Document measurement in chart

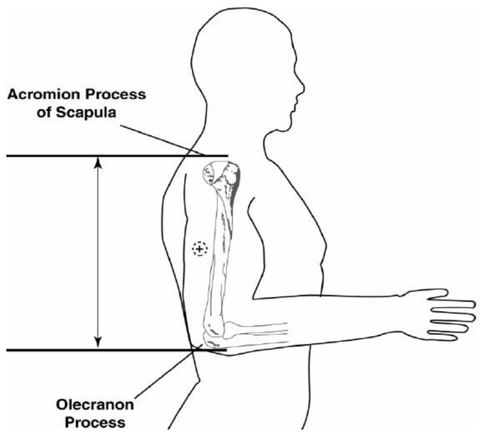

Supplement: Supplementary file 1 [file healthcare-09-00323-s001.zip › S2 Laminated cards.pdf]
