# Supplementary material for: Improving Accurate Blood Pressure Cuff Allocation in Patients with Obesity: A Quality Improvement Initiative
Source: Healthcare (Basel). 2021 Mar 13;9(3):323. doi: 10.3390/healthcare9030323 (PMC8000816; doi:10.3390/healthcare9030323)
Supplement: Supplementary file 1 [file healthcare-09-00323-s001.zip › S3 Staff Questionnaires.pdf]

S3 Staff Questionnaires

Pre-operative Staff

The following questions relate to the practice of allocating blood pressure cuffs to patients who are going to be cared for in the operating theatre complex.

1. There was always a tape measure available for me to use.

Strongly disagree	Disagree	Neither agree nor disagree	Agree	Strongly agree
1	2	3	4	5

2. These was always the correct cuff size available when I needed it.

Strongly disagree	Disagree	Neither agree nor disagree	Agree	Strongly agree
1	2	3	4	5

3. It was easy to measure the arm length and the mid arm circumference.

Strongly disagree	Disagree	Neither agree nor disagree	Agree	Strongly agree
1	2	3	4	5

4. After taking the measurement, it was easy to choose the correct cuff.

Strongly disagree	Disagree	Neither agree nor disagree	Agree	Strongly agree
1	2	3	4	5

5. I feel confident taking the measurement and choosing the correct cuff.

Strongly disagree	Disagree	Neither agree nor disagree	Agree	Strongly agree
1	2	3	4	5

6. Taking these measurements added a lot of extra work for me.

Strongly disagree	Disagree	Neither agree nor disagree	Agree	Strongly agree
1	2	3	4	5

7. Documenting the mid arm circumference added a lot of extra work for me.

Strongly disagree	Disagree	Neither agree nor disagree	Agree	Strongly agree
1	2	3	4	5

8. If you have other information to pass on regarding this clinical practice please include in the space below.

Intraoperative Staff

The following questions relate to your recent experience (over the last month) of caring for obese patients in the operating theatre. Please circle the response which best describes your experience in caring **for obese patients only eg BMI >35 kg/m^2.**

1. The allocated blood pressure cuff is usually acceptable to me.

Strongly disagree	Disagree	Neither agree nor disagree	Agree	Strongly agree
1	2	3	4	5

2. I often need to change the cuff to a different size.

Strongly disagree	Disagree	Neither agree nor disagree	Agree	Strongly agree
1	2	3	4	5

3. I do not usually notice what sized blood pressure cuff has been allocated.

Strongly disagree	Disagree	Neither agree nor disagree	Agree	Strongly agree
1	2	3	4	5

3. If you have other information to pass on regarding this clinical practice please include in the space below.

____

Post-operative Staff

The following questions relate to your recent experience (over the last month) of caring for obese patients in the operating theatre. Please circle the response which best describes your experience in caring **for obese patients only eg BMI >35 kg/m^2.**

1. The allocated blood pressure cuff is usually acceptable to me.

**Strongly disagree**	**Disagree**	**Neither agree nor disagree**	**Agree**	**Strongly agree**
**1**	**2**	**3**	**4**	**5**

2. I often need to change the cuff to a different size.

**Strongly disagree**	**Disagree**	**Neither agree nor disagree**	**Agree**	**Strongly agree**
**1**	**2**	**3**	**4**	**5**

2. I do not usually notice what sized blood pressure cuff has been allocated.

**Strongly disagree**	**Disagree**	**Neither agree nor disagree**	**Agree**	**Strongly agree**
**1**	**2**	**3**	**4**	**5**

3. If you have other information to pass on regarding this clinical practice please include in the space below.

____
